# Comparison of Expansive Pedicle Screw and Polymethylmethacrylate-Augmented Pedicle Screw in Osteoporotic Sheep Lumbar Vertebrae: Biomechanical and Interfacial Evaluations

**DOI:** 10.1371/journal.pone.0074827

**Published:** 2013-09-23

**Authors:** Da Liu, Yi Zhang, Bo Zhang, Qing-yun Xie, Cai-ru Wang, Jin-biao Liu, Dong-fa Liao, Kai Jiang, Wei Lei, Xian-ming Pan

**Affiliations:** 1 Department of Orthopaedics, General Hospital of Chengdu Military Region, Chengdu, Sichuan Province, P.R.China; 2 Affiliated Stomatological Hospital, General Hospital of Chengdu Military Region, Chengdu, Sichuan Province, P.R.China; 3 Department of Orthopaedics, Xijing Hospital, Fourth Military Medical University, Xi′an, Shaanxi Province, P.R.China; Georgia Regents University, United States of America

## Abstract

**Background:**

It was reported that expansive pedicle screw (EPS) and polymethylmethacrylate-augmented pedicle screw (PMMA-PS) could be used to increase screw stability in osteoporosis. However, there are no studies comparing the two kinds of screws *in vivo*. Thus, we aimed to compare biomechanical and interfacial performances of EPS and PMMA-PS in osteoporotic sheep spine.

**Methodology/Principal Findings:**

After successful induction of osteoporotic sheep, lumbar vertebrae in each sheep were randomly divided into three groups. The conventional pedicle screw (CPS) was inserted directly into vertebrae in CPS group; PMMA was injected prior to insertion of CPS in PMMA-PS group; and the EPS was inserted in EPS group. Sheep were killed and biomechanical tests, micro-CT analysis and histological observation were performed at both 6 and 12 weeks post-operation. At 6-week and 12-week, screw stabilities in EPS and PMMA-PS groups were significantly higher than that in CPS group, but there were no significant differences between EPS and PMMA-PS groups at two study periods. The screw stability in EPS group at 12-week was significantly higher than that at 6-week. The bone trabeculae around the expanding anterior part of EPS were more and denser than that in CPS group at 6-week and 12-week. PMMA was found without any degradation and absorption forming non-biological “screw-PMMA-bone” interface in PMMA-PS group, however, more and more bone trabeculae surrounded anterior part of EPS improving local bone quality and formed biological “screw-bone” interface.

**Conclusions/Significance:**

EPS can markedly enhance screw stability with a similar effect to the traditional method of screw augmentation with PMMA in initial surgery in osteoporosis. EPS can form better biological interface between screw and bone than PMMA-PS. In addition, EPS have no risk of thermal injury, leakage and compression caused by PMMA. We propose EPS has a great application potential in augmentation of screw stability in osteoporosis in clinic.

## Introduction

The transpedicular screw system has been widely used in treating degenerative disorders, unstable fractures, deformations and tumors of the spine in the past two decades [1], [2], [3]. However, the screw loosening or failure occurs in cases of inadequate pedicle screw fixation strength or mechanical overload of the repaired spine [4], especially in patients with osteoporosis [5].

Enhancement of pedicle screw stability in osteoporosis can be achieved by using bigger or longer screws [6], [7], [8] and augmenting screw fixation with polymethylmethacrylate (PMMA) bone cement [8], [9], [10], [11]. However, pedicle fracture [12], perforation of anterior vertebral cortex and vascular or visceral injuries may be caused by using bigger or longer screws [13]. A main possible drawback of PMMA augmentation is neurologic injury resulting from cement leakage or thermal effects. To address these issues, some researchers and our group designed an expansive pedicle screw (EPS). It expands radially at the anterior half part of screw in vertebral body and keeps the diameter of the posterior half part of screw unchanged in pedicle. It has already been proved [14], [15], [16], [17], [18], [19] that EPS can significantly improve screw fixation strength and reduce the risk of pedicle penetration in situations of compromised bone quality.

Intriguingly, we found from the related literatures [8], [9], [10], [11] that, in spite of neurologic injury risk, PMMA is widely used for pedicle screw augmentation in osteoporosis due to its distinguished mechanical performance. However, neither biomechanical nor interfacial comparisons of EPS and polymethylmethacrylate-augmented pedicle screw (PMMA-PS) were found, especially in primary spinal instrumentation. It had been proved that, despite specific differences in various morphologic indices, the anisotropy of lumbar cancellous bone in sheep showed the same trend as that of human beings, so sheep lumbar vertebrae may be a good choice for experiments of spinal instrumentation [20], [21]. Therefore, we chose sheep as experimental material *in vivo* in this study. The biomechanical fixation strength of EPS and PMMA-PS was compared through axial pullout tests. The interface between screw and bone tissue was evaluated through micro-computerized tomography (micro-CT) analysis and histological observation.

## Materials and Methods

### Ethics Statement

This study was carried out in strict accordance with the Guidelines for the Care and Use of Laboratory Animals established by National Ethics Committee. All the procedures involving sheep were approved by the Ethics Committee of Animal Experiments of General Hospital of Chengdu Military Region (Permit Number: 1998-0106). All the operations and sacrifices of sheep were performed under general anesthesia with sumianxin at 0.1 ml/kg [22] (a complex prescription composed of xylidinothiazoline, edathamil, etorphine and aloperidin, Military Veterinary Institute, Veterinary University of PLA, Beijing, China), and all efforts were made to minimize suffering.

### Implant Materials

**Figure 1:**
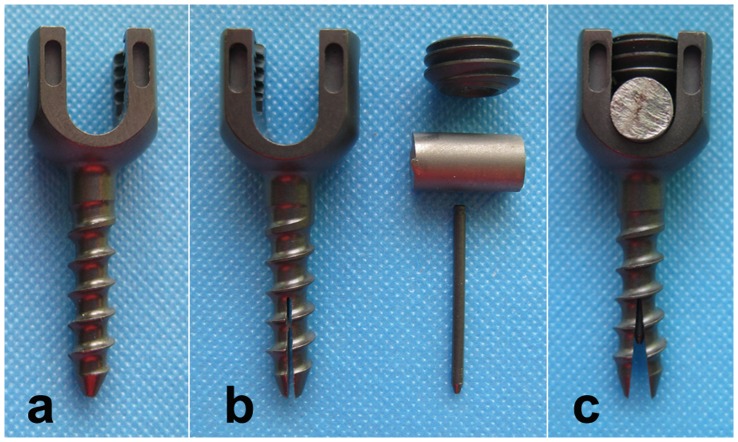
The different kinds of pedicle screws used in this study. “a” is the conventional pedicle screw (CPS). “b” is the component elements of expansive pedicle screw (EPS), including EPS, bolt, rod and nut. “c” is the expanding EPS, which was achieved through inserting the bolt into the interior of EPS, pressing the rod onto end of the bolt and tightening the nut into the end of screw.

The conventional pedicle screws (CPS) are identical with a length of 20.0 mm and an outer diameter of 4.5 mm (Figure 1a). The expansive pedicle screws (EPS) have a length of 20.0 mm, an outer diameter of 4.5 mm and a diameter-1.0 mm internal bore (Figure 1b). The anterior half portion of the screw was split lengthwise by a groove to form two anterior fins. A smaller gauge bolt with diameter of 1.0 mm was inserted into the interior of the EPS in order to open the fins at tip of EPS (Figure 1c). It is designed that when the bolt is taken out the anterior expanded part will be retracted to some extent during the removing of EPS. This system increases the diameter of the anterior half part of the EPS, while the diameter of the posterior portion of the screw remains constant. Both CPS and EPS (Medtronic-Weigao Orthopedic Device Company, Shandong, China) were made of titanium alloy and designed only for sheep lumbar vertebrae. 64 conventional pedicle screws and 32 expansive pedicle screws were used in this study. For screw augmentation, surgical PMMA (CEMEX, TECRES, Verona, Italy) was used.

### Induction of Osteoporosis Animal Model

Eight female Chinese white sheep (age 5.5±0.7 years, mean body weight 55.7±5.6 kg) underwent bilateral ovariectomy (OVX) and received intramuscular injection of 0.45 mg/kg body weight methylprednisolone [23], [24] (Pfizer Pharmaceutical Co., Ltd, USA) per day beginning 4 weeks after OVX. Steroid administration was continued for 10 months. In the last 4 weeks, the dosage was slowly reduced from full dose, to half dose, 1/4 dose, 1/8 dose…and finally to zero to avoid withdrawal symptoms caused by abrupt end of high-dose methylprednisolone [23], [24]. After the cessation of the methylprednisolone administration, the animals were kept for another one month for observation of adverse reaction. The BMD values of sheep lumbar vertebrae was measured using a dual-energy X-ray absorptiometry (Lunar Corp., Madison, WI, U.S.A.) before OVX and after induction of osteoporosis and were recorded as BMD pre-induction and BMD post-induction respectively. In this study, more than 25% decrease in BMD of lumbar spine was considered as successful induction of osteoporotic sheep [25].

### Experimental Procedures

All sheep were anesthetized with sumianxin at 0.1 ml/kg and placed at prone position on a four-poster frame. Each lamina from the first lumbar vertebra (L1) to the sixth lumbar vertebra (L6) was exposed by the posterior midline approach. Six lumbar vertebrae were divided into three groups randomly (two vertebrae with four pedicles in each group). In all groups, the screw insertion point was at the intersection of the upper 1/3 line of transverse process and the vertical tangential line of the lateral margin of the zygapophyseal joint. The pilot hole was prepared 20.0 mm in depth with a 2.5-mm drill in both pedicles of each vertebra, following an angle of 40° to the spinous process in the sagittal plane. The hole was then checked with a probe to ensure that the pedicle had not been violated. Each hole was then tapped using a 3.0-mm tap (Medtronic-Weigao Orthopedic Device Company, Shandong, China). The hole was again checked with a probe to verify an intact pedicle. In CPS group, CPS was inserted through the pilot hole into vertebral body without any augmentation. In PMMA-PS group, PMMA (1.0 ml) was injected into the pilot hole prior to the insertion of CPS. The bone cement powder was mixed with the cement solution at ratio of 2∶1 (gram to milliliter) according to manufacturer’s recommendations. The cement was injected in the toothpaste-like phase. In EPS group, EPS was inserted through pedicle into vertebral body. All screws were inserted by hand using a ratcheted screwdriver (Medtronic-Weigao Orthopedic Device Company, Shandong, China) until the hub of the screw was firmly seated against the posterior cortex. The incision was sutured by layers after insertion of all screws. A medicine (cefazolin sodium, 2.0 mg/d) for the prevention of injection was administrated through intramuscular injection for five days. Eight sheep were randomized into two study periods of 6 weeks post-operation (n = 4) and 12 weeks post-operation (n = 4). The sheep were sacrificed and lumbar vertebrae (L1-L6) were harvested at the end of the assigned study periods. For two pedicle screws in each vertebra, one side was selected randomly for axial pullout tests. After biomechanical tests, the contralateral side was prepared for micro-CT analysis and histological evaluation of screw-bone interfacial properties.

### Axial Pullout Tests

**Figure 2:**
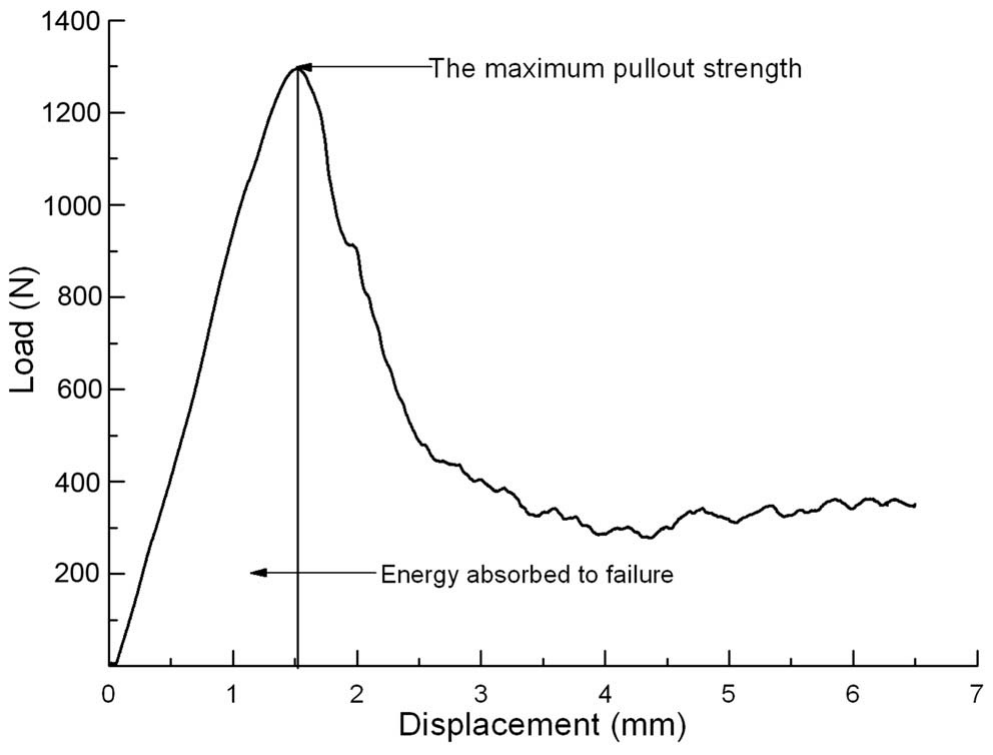
The load-displacement curve in the axial pullout test. On the curve, the maximum pullout strength (F_max_) was defined as the inflection point where the load peaked and then sharply decreased with the increasing displacement and energy absorbed to failure was determined as the area under the curve before the onset of failure point.

The screw-placed vertebrae were mounted onto a special jig, which allowed for rotation about the x- and z-axis. The pedicle screw was withdrawn upwards through a rotation-free connection (including y-axis) to the hydraulic actuator. The customized jig and the rotation-free connection ensured an axial pullout without bending of the screw. Once the specimen was tightly secured, each screw was pulled at a constant speed of 5 mm/min [26], [27], [28] by MTS 858 Material Testing System (MTS System, Minneapolis, MN, USA) until purchase failure. Load and displacement data were obtained in real time at 50 Hz and used to obtain load-displacement curve. On the curve, the maximum pullout strength (F_max_) was defined as the inflection point where the load peaked and then sharply decreased with the increasing displacement and energy absorbed to failure was determined as the area under the curve before the onset of failure point (Figure 2) [18], [22].

### Micro-CT Analysis

The vertebrae were sawed into a cylinder-shaped sample using the special-designed trephine. The sample contained screw in the center with bone tissues surrounding the screw and had a total diameter of 1.5 cm. Micro-CT scanning was carried out at a resolution of 21 µm with the eXplore Locus SP Micro-CT system (Healthcare, GE, USA). Because of the characteristic CT values, screw, PMMA and bone trabeculae could be respectively reconstructed by MicroView software (Healthcare, GE, USA), which then was helpful in observing the interface between bone and screw in each group.

**Figure 3:**
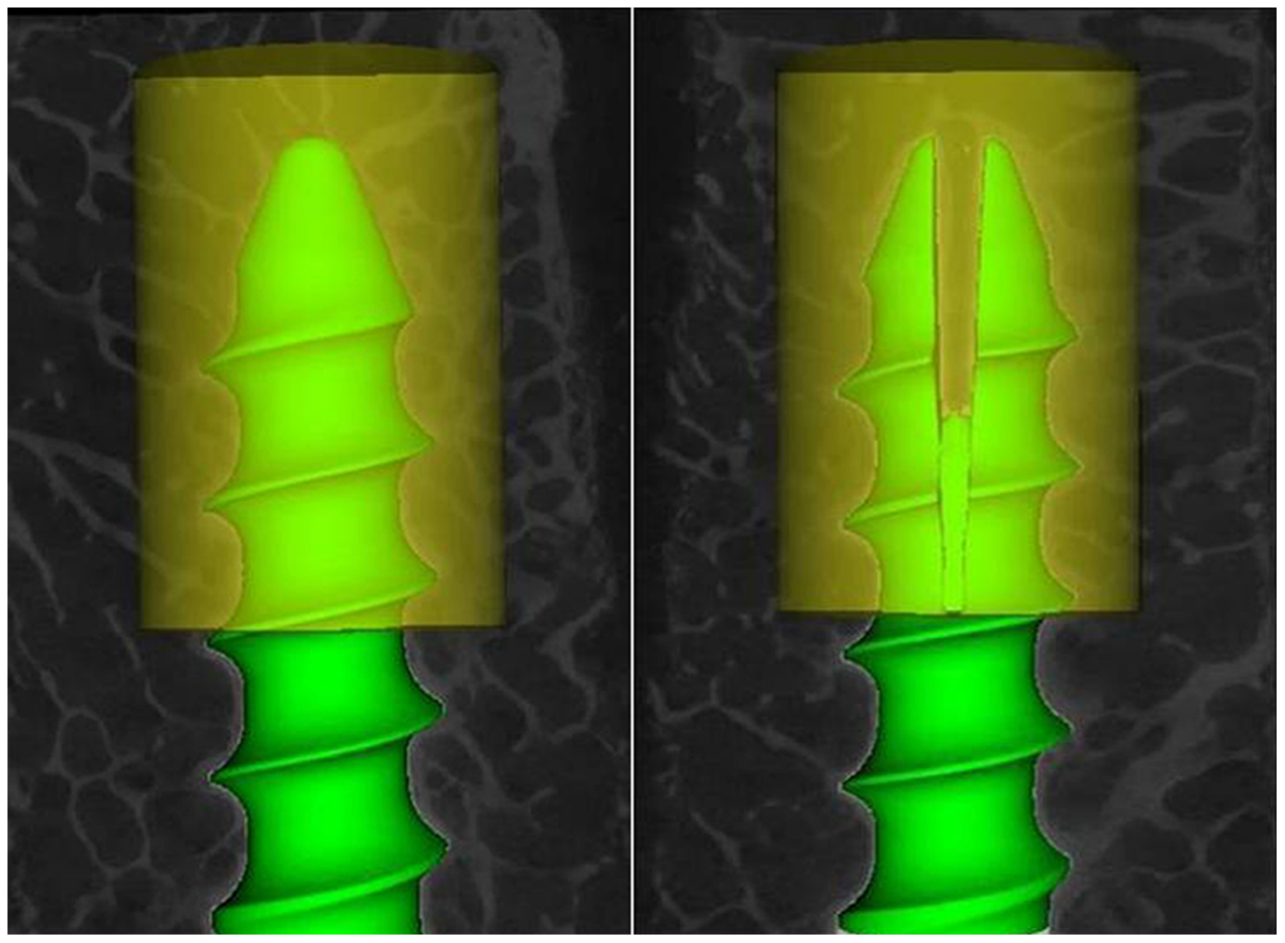
ROI of anterior part of screw. Left was CPS and right was EPS. ROI indicates regions of interest.

Histomorphometric analysis of bone tissue was performed in the same region of interest (ROI) around the anterior half part of pedicle screw in both CPS and EPS groups (Figure 3) with the same threshold (CT value >1000) using Advanced Bone Analysis software (GE Healthcare, USA). The main spatial structure parameters, such as tissue mineral density (TMD, mg/cm^3^), bone volume/total volume (BVF, %), bone surface/bone volume (BS/BV, mm^−1^), trabecular thickness (Tb.Th, µm), trabecular number (Tb.N, mm^−1^) and trabecular spacing (Tb.Sp, µm) were determined.

### Histological Observations

After micro-CT scanning, the same specimens were prepared for histological observation. The specimens were fixed in 10% phosphate-buffered formalin pH 7.25 for 7 days. Then they were dehydrated with increasing concentrations of ethanol (70%, 80%, 90%, 99%, 100%, and 100% v/v) for 18 hours at each concentration. Specimens were then embedded in polyester resin and kept for 3 weeks. Continuous four sections with thickness of 30∼50 µm were cut with a band saw (Leica-LA 2500, Germany) strictly perpendicular to the anterior half part of screw approximately from the screw tip. Sections were then stained with modified Ponceau trichrome staining. A thorough microscopic analysis was performed using a transmitted light microscope (Leica-LA, Germany) combined with a digital camera (Pixera Pro600cl, USA).

### Statistic Analysis

Statistical analysis was performed with SPSS 16.0. The data were expressed as means ± SD. Through Shapiro-Wilk test and Levene test, all the data showed the normal distribution and the biomechanical data had the homogeneity of variance. Paired t test was used to compare BMD pre-OVX and post-OVX. The one-way ANOVA was used to detect the differences in F_max_ and E among the three groups and the SNK-q test was used to compare data between any two groups. Independent t test was used to compare biomechanical data between two study periods in all groups and compare the spatial structure parameters between CPS and EPS groups at same study period and between two study periods in same group. P<0.05 was considered statistically significant.

## Results

### General Observation of Sheep

There was no complications detected post-OVX. All incisions were healed well. No infections of injection point and no obvious side effects of methylprednisolone were found during 12-month induction of osteoporosis. After injection of PMMA and insertion of screw, there were no any neurologic complications caused by leakage of PMMA or malposition of screw. All incisions were healed well.

### Measurement of BMD

Twelve months after ovariectomy, the mean BMD value of sheep lumbar vertebrae post-induction was significantly lower than pre-induction (p<0.001) (Table 1).

**Table 1:** BMD of lumbar vertebrae pre-induction and post-induction (n = 8, means ± SD).

BMD (g/cm^2^)	1	2	3	4	5	6	7	8	Mean
Pre-induction	1.16	1.19	1.28	0.97	1.05	1.14	1.08	1.25	1.14±0.10
Post-induction	0.84	0.86	0.91	0.72	0.77	0.85	0.80	0.92	0.83±0.07*
Decrease (%)	27.6	27.7	28.9	25.8	26.7	25.4	25.9	28.0	27.2

BMD indicates bone mineral density;

* Compared with Pre-induction, p<0.05.

### Axial Pullout Tests

At 6-week and 12-week, F_max_ and E in both EPS and PMMA-PS groups were significantly higher than that in CPS group (p<0.05). There were no significant differences on both F_max_ and E between PMMA-PS and EPS groups at both 6-week and 12-week (p>0.05). There were also no significant increments on F_max_ and E in CPS and PMMA-PS groups between 6-week and 12-week (p>0.05). In EPS group, however, F_max_ and E at 12-week was approximately 21.3% and 24.6% higher than those at 6-week respectively (p = 0.037 and 0.035, respectively) (Table 2).

**Table 2:** Results in axial pullout tests in three groups (n = 8, means ± SD).

Study periods	Parameters	CPS group	PMMA-PS group	EPS group
6-week	F_max_ (N)	827.88±139.22	1426.38±235.75*	1252.13±203.51*#
	E (J)	1.66±0.30	2.84±0.55*	2.48±0.45*#
12-week	F_max_ (N)	906.63±152.50 &	1472.75±248.65*&	1518.88±256.81*#Δ
	E (J)	1.80±0.35 &	2.95±0.60*&	3.09±0.59*#Δ

CPS, PMMA-PS and EPS indicate conventional pedicle screw, polymethylmethacrylate-augmented pedicle screw and expansive pedicle screw, respectively. F_max_ and E indicate the maximum pullout strength and energy-to-failure, respectively.

* Compared with CPS group at the same study period, p<0.05; # Compared with PMMA-PS group at the same study period, p>0.05; & Compared with 6-week in the same group, p>0.05; Δ Compared with 6-week in the same group, p<0.05.

### Micro-CT Analysis

**Figure 4:**
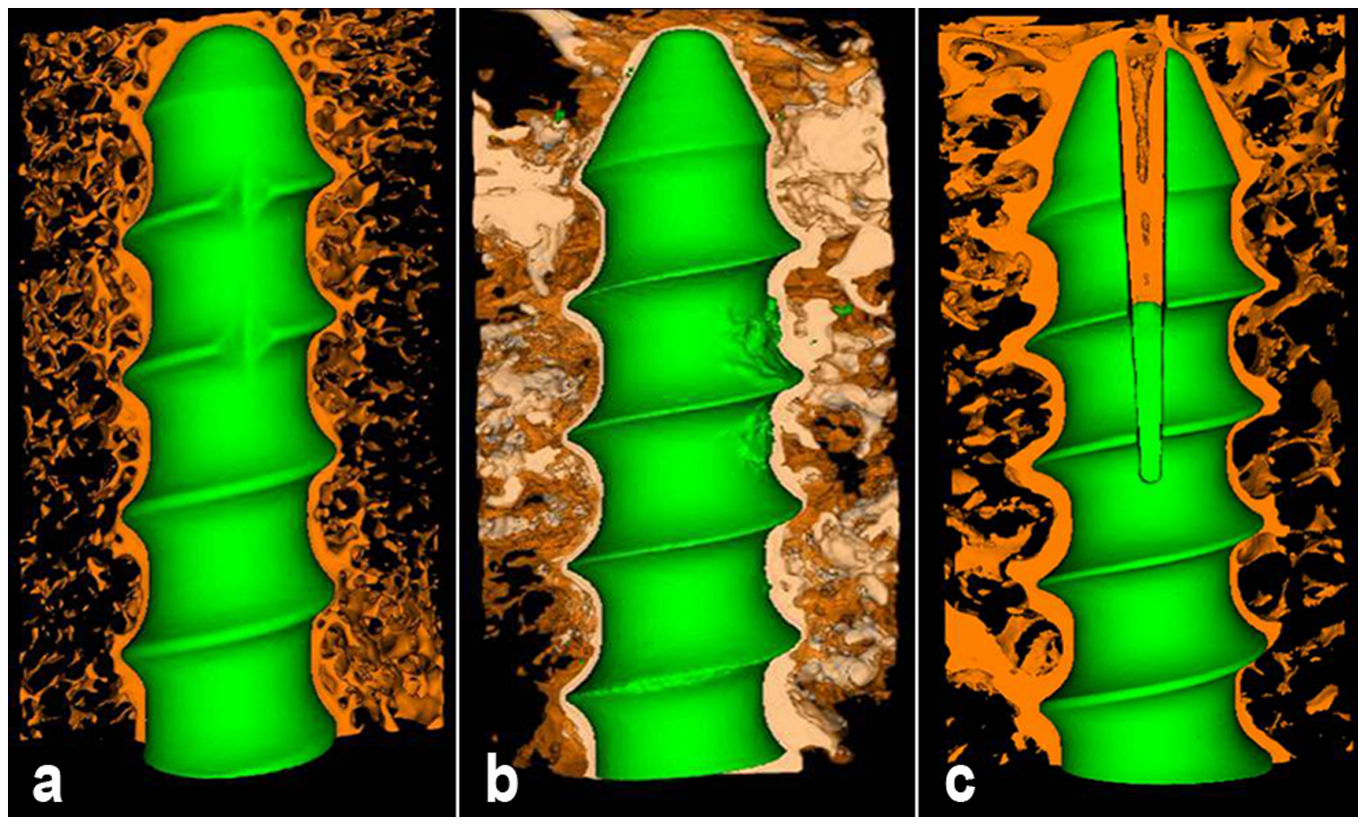
Three-dimension micro-CT reconstruction of interface between bone and screw in three groups at 6-week. “a”, “b” and “c” indicate CPS group, PMMA-PS group and EPS group, respectively. Brown color, blue color and white color represent bone tissue, screw and PMMA, respectively. (Resolution 21 µm, 2048×2048).

**Figure 5:**
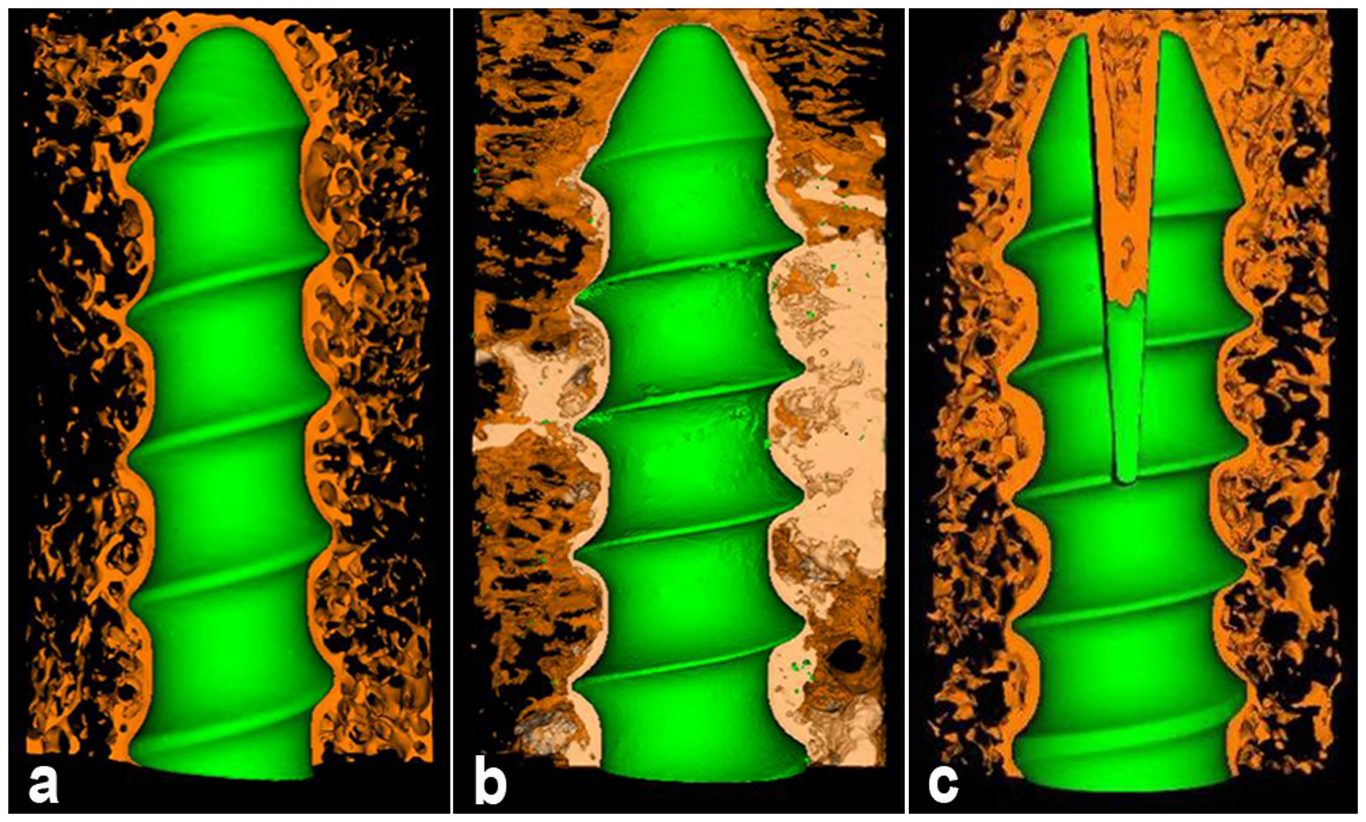
Three-dimension micro-CT reconstruction of interface between bone and screw in three groups at 12-week. “a”, “b” and “c” indicate CPS group, PMMA-PS group and EPS group, respectively. Brown color, blue color and white color represent bone tissue, screw and PMMA, respectively. (Resolution 21 µm, 2048×2048).

At both 6-week and 12-week, bone trabeculae wrapped up screw directly forming “screw-bone” interface in CPS and EPS groups (Figure 4a, 5a, 4c, 5c). PMMA surrounding the screw hampered the direct contact between bone and screw and formed “screw-PMMA-bone” interface in PMMA-PS group (Figure 4b, 5b). The expanding part of EPS pressed surrounding bone trabeculae and made local bone tissue visually more and denser than those around anterior half part of screw in CPS group (Figure 4c, 5c). There was no visual difference in amount and spatial structure of bone trabeculae around screw between 6-week and 12-week in both CPS and PMMA-PS groups (Figure 4, 5). Nevertheless, bone trabeculae around expanding part of EPS at 12-week were visually more and denser than that at 6-week (Figure 4c, 5c).

The metrological analysis demonstrated that TMD, BVF, Tb.Th, Tb.N in EPS group were significantly higher than those in CPS group and BS/BV, Tb.Sp in EPS group were significantly lower than those in CPS group at both 6-week and 12-week (p<0.05). There were no significant differences on all parameters between 6-week and 12-week (p>0.05) in CPS group. Except BS/BV, nevertheless, TMD, BVF, Tb.Th, Tb.N significantly increased and Tb.Sp significantly decreased at 12-week compared with those at 6-week in EPS group (p<0.05) (Table 3).

**Table 3:** Three dimensional parameters of RIO in CPS and EPS groups (n = 8, means ± SD).

Parameters	6-week		12-week	
	CPS group	EPS group	CPS group	EPS group
TMD (mg/cc)	387.46±40.74	447.83±43.13 *	423.43±46.89	506.06±50.27 *#
BVF (%)	44.05±7.46	57.05±8.37 *	51.92±8.24	67.55±9.40 *#
BS/BV (mm^−1^)	15.02±3.51	11.01±2.31 *	12.05±2.71	8.90±1.82 *
Tb.Th (µm)	153.84±25.68	227.85±38.27 *	184.57±36.96	276.24±48.19 *#
Tb.N (mm^−1^)	2.11±0.22	2.53±0.28 *	2.35±0.29	2.90±0.34 *#
Tb.Sp (µm)	281.18±39.09	234.37±30.73 *	257.18±34.45	201.25±25.61 *#

ROI indicates region of interest, CPS and EPS indicate conventional pedicle screw and expansive pedicle screw, respectively.

* Compared with CPS group at the same study period, p<0.05; # Compared with 6-week in the same group, p<0.05.

### Histological Observations

**Figure 6:**
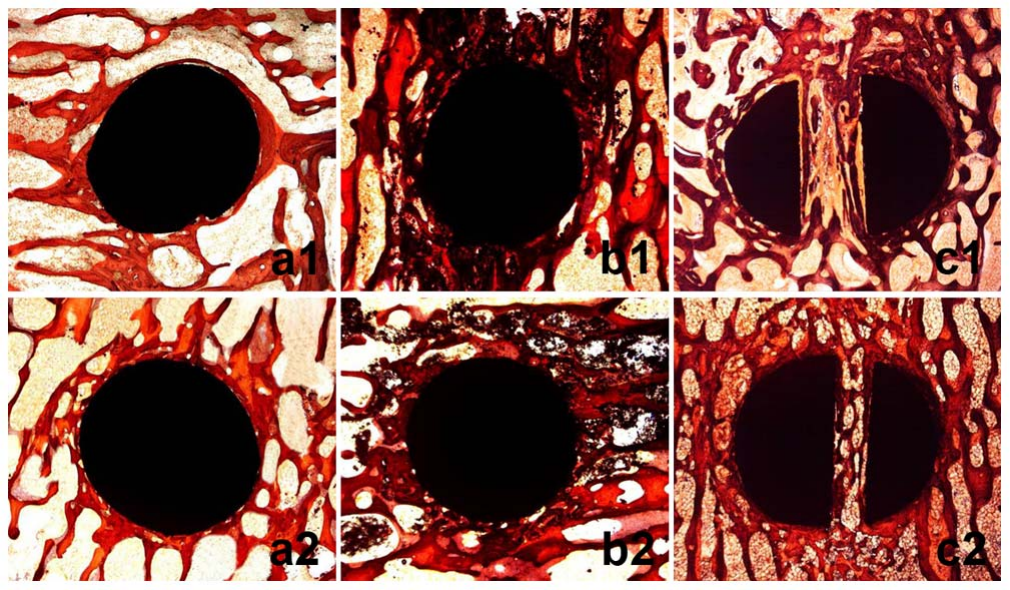
Histological observations of interface between bone and screw in three groups at 6-week and 12-week. “a”, “b” and “c” indicate CPS group, PMMA-PS group and EPS group, respectively. “1″ and “2″ indicate16 and 50 times magnification in microscope, respectively. Red color and saffron yellow color represent mature bone trabeculae and fibrous tissue, respectively. Round black and irregularly-shaped black colors represent screw and PMMA, respectively.

In CPS group (Figure 6a1, 6a2), bone trabeculae wrapped up the screw directly and formed “screw-bone” interface. There was no visual difference on the quality of bone tissue around screw (amount and density of bone tissue) between 6-week and 12-week. In PMMA-PS group (Figure 6b1, 6b2), PMMA surrounding the screw hampered the direct contact between bone and screw and formed “screw-PMMA-bone” interface. At both 6-week and 12-week, PMMA was found between bone and screw and in cavitas medullaris around screw, which caused unclear circumscription between bone tissue and screw. No visual difference on the quality of bone tissue around screw was found between 6-week and 12-week. From 6-week to 12-week, PMMA was found remaining around screw without any degradation and absorption, which led to the non-biological interface. In EPS group (Figure 6c1, 6c2), bone trabeculae wrapped up screw formed “screw-bone” interface. The anterior part of screw expanded obviously and pressed bone tissue, and the bone trabeculae were more and denser than those in CPS group at both 6-week and 12-week. From 6-week to 12-week, more and more bone trabeculae surrounded screw, crept into interspaces between two fins and connected with each other forming mature structure of cavitas medullaris around screw. At 12-week the mature bone tissue took place of the fibrous tissue at 6-week and formed biological interface, which improved the quality of bone tissue around anterior part of screw.

## Discussion

Many methods were developed to increase screw fixation strength in quality-deficient bone tissue. PMMA has been shown to be the most available, cost effective, and strongest material for augmentation and been used in many clinical orthopedic applications for decades [3], [8], [9], [10], [11]. Cook [18] reported that PMMA injection through a specially designed expanded screw increased pullout strength by 250% in severely osteoporotic bone, compared with the non-cemented expandable screw. The results showed that PMMA could make notable contributions to augmentation of screw stability in osteoporosis.

However, to our knowledge, there were few studies about biomechanical and interfacial comparisons between EPS and PMMA-PS, especially in primary spine instrumentation in osteoporosis. In a pilot research [17], we found that EPS could markedly enhance screw stability with a similar effect to the traditional PMMA-PS in primary surgery in osteoporotic cadaveric lumbar vertebrae. Due to the osseointegration between screw and bone tissue *in vivo*, the stability and interfacial comparisons of EPS and PMMA-PS will be detected in osteoporotic animal spine. Thus, we induced osteoporosis sheep model firstly and then performed this comparative study in osteoporotic sheep lumbar vertebrae.

In our previous study [29], we found significant reduction on BMD, micro-architectural parameters and mechanical properties one year after only OVX. There was 23% decrease on BMD of lumbar vertebrae between pre-OVX and post-OVX. In order to ensure the successful induction of osteoporosis model, therefore, we prolonged the injection period of steroids to 10 months in this study and considered more than 25% decrease in lumbar BMD as the standard of model establishment in this study. With 12-month OVX and 10-month glucocorticoid-application, there was mean 27.2% decrease on BMD of lumbar vertebrae between pre-induction and post-induction.

In the present study, micro-CT analysis and histological observation revealed that the expanded anterior part of EPS formed a clawlike structure holding bone tissues firmly. The two fins of expanding part performed strong stressing to surrounding bone tissues, which induced the local bone growth and improved the quality of bone tissue (amount and dense of bone trabeculae). The clawlike structure and the surrounding denser bone tissues interacted with each other to maintain the stabilization of screw in theory and it was proved in biomechanical tests that EPS showed higher fixation strength than CPS at both 6-week and 12-week. With the continuous stress stimulation of expanded anterior part from 6-week to 12-week, there was a significantly improvement on the quality of local bone tissue (amount and dense of bone trabeculae) in micro-CT analysis. The fibrous tissue at 6-week were replaced by the mature bone tissue connected with each other forming the cavitas medullaris and the better biological interface at 12-week. Based on the improved bone quality, EPS showed a further significantly improved stability from 6-week to 12-week.

There were no degradation and absorption of PMMA and no significant change on screw stability from 6-week to 12-week. Due to the property of nondegradation, however, PMMA existed around screw and hampered the direct contact between bone and screw for long term. It led to no biointegration of bone and screw and formed the non-biological interface, which may influence the long-term stability of screw. However, it needs the further study on screw stability at the longer study periods post-operation.

However, there were several limitations in present study. It is more scientific and rigorous to monitoring BMD of sheep lumbar vertebrae after insertion of screw until the sacrifice of sheep. In the future study, we will measure the BMD of proximal femur after insertion of screw in spine to assess the change on bone quality. The significant change on sheep bone quality would bring significant influence on screw stability in vivo. As showed in micro-CT reconstruction, PMMA interdigitated with surrounding trabecular bone. It is very difficult to distinguish them completely using density slicing in histomorphometric analysis of bone tissue, which possibly brings bias to our results. Therefore, we just analyzed histomorphometry of bone tissue around EPS and CPS, which may accurately reflect the stabilization mechanism of EPS compared with CPS through histomorphometric analysis. It also did not affect the observations on the interface between bone tissue and screw in all groups. We will connect screws with rod and transverse connection to mimic the biomechanical environment in spinal posterior instrumentation in next research. The same kind of screws will be inserted in one sheep lumbar vertebrae, which is similar to the real condition in clinic. As we found in preliminary study in vitro, three was significant different fixation strength between CPS and EPS and between CPS and PMMA-PS. If we inserted these different kinds of screws in one sheep and connected these screws together with rod and transverse connection, the loading distributed along two rods and transverse connection into all screws and the stress concentration would happen to the screws with lower fixation strength. In this condition, loosening may firstly happen to the screws with lower stability, which would lead to failure of whole structure. We will also prolong the study periods post-operation in order to evaluate the long-term screw stability and observe the long-term interface between screw and bone tissue, which will be more similar to the real condition of follow up in clinic and be more helpful to our clinical assessments. There was just qualitative assessment in histological observation in present study. Therefore, we will perform fluorescence double labeling with tetracycline and calcein in order to evaluate the bone growth in vivo through objective quantitive parameters. It will be more objective to demonstrate the condition of bone growth through several parameters.

In this study, we proved that EPS could markedly enhance screw stability with a similar effect to the traditional method of screw augmentation with PMMA in initial surgery in osteoporosis. EPS shows excellent early stability and interface in vivo. In addition, EPS have no risk of thermal injury, leakage and compression caused by PMMA. In conclusion, we propose that EPS is an effective, safe and easy method and has a great application potential in augmentation of screw stability in osteoporosis in clinic.
